# Evaluating the social benefits and network costs of heat pumps as an energy crisis intervention

**DOI:** 10.1016/j.isci.2024.108854

**Published:** 2024-01-11

**Authors:** Yihong Zhou, Chaimaa Essayeh, Sarah Darby, Thomas Morstyn

**Affiliations:** 1 School of Engineering, University of Edinburgh, Edinburgh EH9 3FB, UK; 2 Environmental Change Institute, University of Oxford, Oxford OX1 3QY, UK; 3 Department of Engineering Science, University of Oxford, Oxford OX1 3PJ, UK

**Keywords:** Energy management, Energy modeling, Energy systems, Social sciences

## Abstract

Fuel poverty, a pressing issue affecting social prosperity, has been exacerbated during the energy crisis triggered by the Russia-Ukraine conflict. This problem can be more severe for off-gas regions. Our study investigates heat pumps (HPs) as a cost-effective alternative to off-gas heating to alleviate fuel poverty in England and Scotland. We analyze regional fuel poverty rates and the associated greenhouse gas emission reduction by replacing all off-gas heating with HPs, observing positive effects under pre-crisis and crisis conditions, with existing government support for HP upfront costs. HP rollout can burden distribution networks especially for certain regions, but our correlation analysis shows that high benefits do not always come with network costs at the regional level, and we identify “priority” regions with low costs and high benefits. These findings provide valuable insights for policymakers to address fuel poverty and reach decarbonization. The methodology is adaptable to other countries with appropriate datasets.

## Highlights

• Replacing off-gas heating with heat pumps reduces fuel poverty in England and Scotland
• Government support is necessary to offset the high upfront costs of heat pumps
• For specific regions heat pumps offer high benefits with low network upgrade costs

## Graphical abstract

**Figure:**
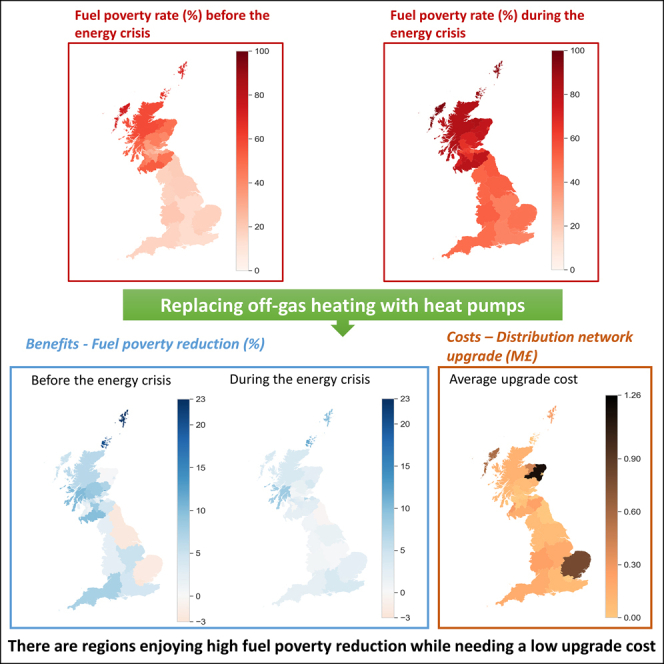

## Introduction

The COVID-19 pandemic and the Russia-Ukraine conflict have led to soaring energy prices and subsequent inflation.^1^^,^^2^ This crisis has intensified concerns about fuel poverty, a state where households struggle to afford the energy needed to heat their homes comfortably.^3^ The far-reaching consequences of fuel poverty on households and society include physical and mental health issues,^4^ increased financial demands on national health systems,^5^ and environmental implications due to the energy inefficiency of fuel-poor homes.^6^ In the UK, energy bills have reached unprecedented levels, with an alarming two-thirds of households at risk of experiencing fuel poverty by January 2023.^7^ Consequently, addressing fuel poverty is vital for social development and prosperity.^8^

A concurrent global trend is the pursuit of decarbonization targets. In the UK, the goal is to achieve net-zero emissions by 2050,^9^ with heating electrification playing a critical role in reaching this target.^10^ Currently, around 11% of UK homes are electrically heated, of which roughly half are heated by electric storage heaters (ESHs) and half by electric direct resistance heaters (ERH).^11^ With appropriate (economic) incentives, ESH can operate flexibly and reduce the burden on power system infrastructure.^12^ However, currently deployed electric heating is still costly and is associated with the highest risk of fuel poverty among all main heating fuels.^13^^,^^14^ Heat pumps (HPs) offer approximately three times the efficiency of resistive electric heaters,^15^ meaning the same heating requirement can be met with one-third of the energy consumption. This efficiency roughly offsets the additional cost of electricity compared to the cost of gas. It should be noted that UK electricity prices are closely coupled with gas prices due to gas being the main generation source for electricity in the UK (around 32% in 2023^16^) and the marginal pricing mechanism;^17^ previous studies have demonstrated the economic benefits of HPs,^15^^,^^18^ revealing costs comparable to mains gas systems and making HPs particularly attractive to off-gas households. However, it is also important to consider how the reduced operational costs of HPs can be linked to social metrics (fuel poverty) related to residents’ quality of life.

Widespread heating electrification also places considerable strain on the energy system infrastructure. Previous research has explored how HPs can increase national electricity demand^19^^,^^20^^,^^21^ and impact the distribution grid infrastructure.^22^ Deetjen et al.^23^ analyzed the economic benefits and increased electricity demand for US cities. However, there is a lack of work that simultaneously examines HP replacement as a means of alleviating fuel poverty and its associated electricity network upgrade costs, which additionally considers the network capacity headroom rather than the demand increase only.

Addressing this gap, our work provides a comprehensive analysis of the potential benefits of HPs replacing the off-gas heating in reducing fuel poverty and greenhouse gas (GHG) emissions while assessing the associated energy distribution network upgrade costs at a regional level. Using processed national household datasets from England and Scotland, as well as electricity substation information from the UK Distribution Network Operators (DNOs), we conduct a detailed examination of the 9 government regions in England and the 32 Local Authorities (LAs) in Scotland. Our findings offer valuable insights for policymakers working to address fuel poverty and decarbonization targets, and our method can be adapted to other countries with appropriate datasets.

## Results

In this section, we first exhibit our estimation of fuel poverty rates, followed by the benefits of HPs including fuel poverty reduction and emission reduction. We then estimate the network upgrade cost triggered by the HP rollout and finally a cost-benefit analysis for HPs that integrates the results.

### Fuel poverty estimation

Fuel poverty is a pressing issue that affects a substantial portion of the UK population. Households experiencing fuel poverty struggle to pay the energy needed to maintain a healthy and comfortable indoor temperature.

**Figure 1:**
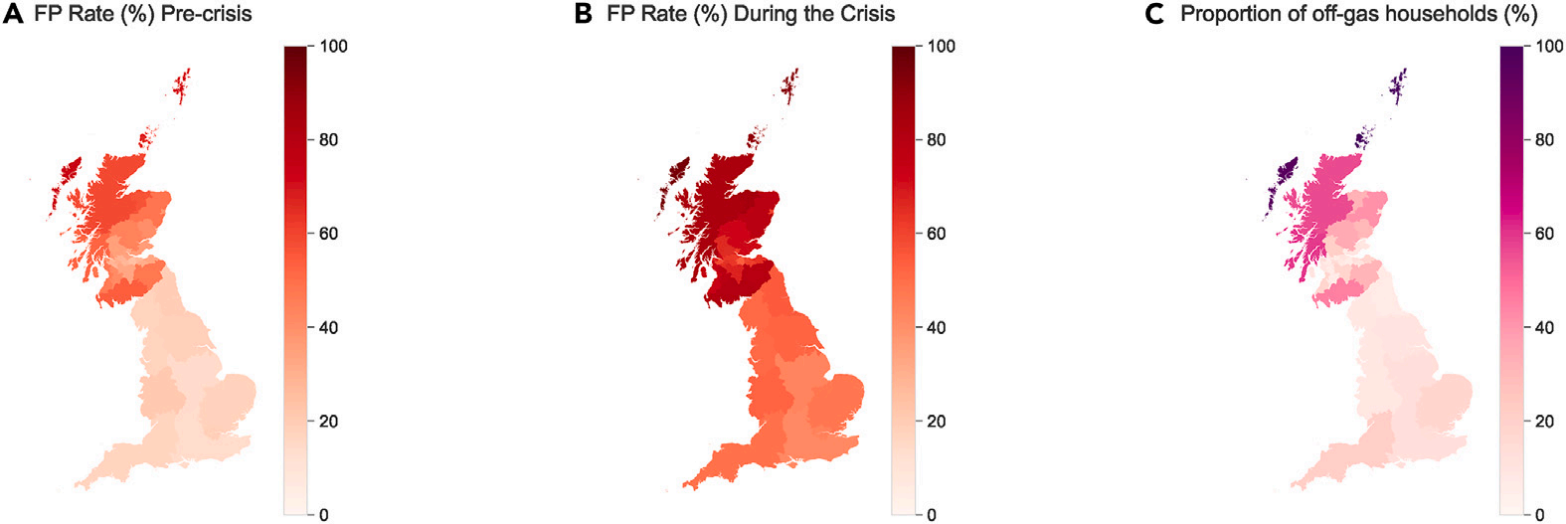
Fuel poverty estimations and proportions of off-gas households (A) Fuel poverty (FP) rate (%) estimation before the energy crisis. (B) FP rate (%) estimation during the energy crisis. (C) The proportion of households using off-gas heating options.

By utilizing government household datasets and energy price data (see Section Household Dataset), we estimate the fuel poverty rates for Scotland and England as presented in Table 1 (‘Before HP’). Here a household is considered fuel-poor if its necessary fuel cost is more than 10% of the household adjusted net income. This is the first condition in Scotland’s definition and is also the one used in Wales (with different calculations for income). A more detailed discussion is given in section method for fuel poverty estimation. The findings indicate that the number of fuel-poor households has nearly doubled due to the energy crisis. Figure 1 offers a regional perspective on the estimated fuel poverty rates, demonstrating that some areas are especially susceptible to fuel poverty. Note that Scotland regions show a higher variation in fuel poverty rates and some regions see higher fuel poverty rates than England, which may be attributed to the lower temperature in Scotland, the finer geographical resolution for Scotland’s results (32 regions compared to 9 regions in England), and the higher proportion off-gas households as Figure 1C. Also, while there have been government estimations of fuel poverty at a more granular regional level,^14^ we conduct our estimations to ensure consistency with our later fuel poverty analysis using our scenarios, i.e., the fuel poverty rates when replacing off-gas heating with HPs in both pre-crisis and crisis periods. Such analysis requires household-level datasets. The publicly available versions of these do not disclose household geographical information lower than the government region level for England and the LA level for Scotland as our Figure 1. Throughout this paper, 2019 prices are considered pre-crisis levels, and 2022 prices are utilized for the estimation during the energy crisis.

**Table 1:** Fuel poverty rate (%) estimation

Period	Country	Scenario	Estimated Fuel Poverty Rate (%)
Pre-crisis	Scotland	Before HP	35.55
Pre-crisis	Scotland	After HP	33.06
Pre-crisis	England	Before HP	17.38
Pre-crisis	England	After HP	16.67
Crisis	Scotland	Before HP	67.26
Crisis	Scotland	After HP	65.16
Crisis	England	Before HP	47.68
Crisis	England	After HP	46.85

To delve deeper into the fuel poverty issue, we explore the proportion of off-gas households in each region, as illustrated in Figure 1C. Intriguingly, the fuel poverty rate heatmap exhibits a pattern similar to the off-gas proportions. The Pearson correlation coefficient between the off-gas proportion and the pre-crisis (respectively over-crisis) fuel poverty estimation is 0.81 (respectively 0.72). The primary reason could be the higher cost of off-gas heating options such as electricity, heating oil, and liquefied petroleum gas (LPG). Note that even though HPs are a form of electric heating, as of the 2022 winter, only 1% of UK households were using HPs as their primary heating method;^24^ hence we assume a baseline HP adoption rate of zero throughout this paper. This finding is also reported in the official fuel poverty reports in England^14^ and Scotland^13^ respectively. Consequently, eco-friendly and economically viable heating technologies are of utmost importance for off-gas households.

### Fuel poverty reduction by heat pumps

HPs are recognized as a cost-effective heating option due to their high heating efficiency. The HP efficiency varies across a year in response to the change in ambient temperature. As the fuel poverty evaluation focuses on the annual energy bill, the Seasonal Performance Factor (SPF) is a better metric to measure the overall efficiency of HPs over a year. Different sources have reported different estimations of the SPF for HPs. The UK Renewable Heat Incentive (RHI) scheme has an average design SPF of 300% for air-source HPs in 2018,^25^ which increases to 360% for new installations in 2022.^26^ A recent HP field trial observed an SPF at 280% for air-source HPs,^27^ showing a significant increase compared to past field trials and a good performance even for cold days. Based on these data, we anticipate that the coming HP rollout can reach an SPF at 300% for both the England and Scotland regions in our subsequent case studies. Furthermore, our later focus will be air-source HPs only, due to their broader applicability than ground-source HPs as analyzed in.^28^ The UK future energy scenario also holds the same expectation.^10^

**Figure 2:**
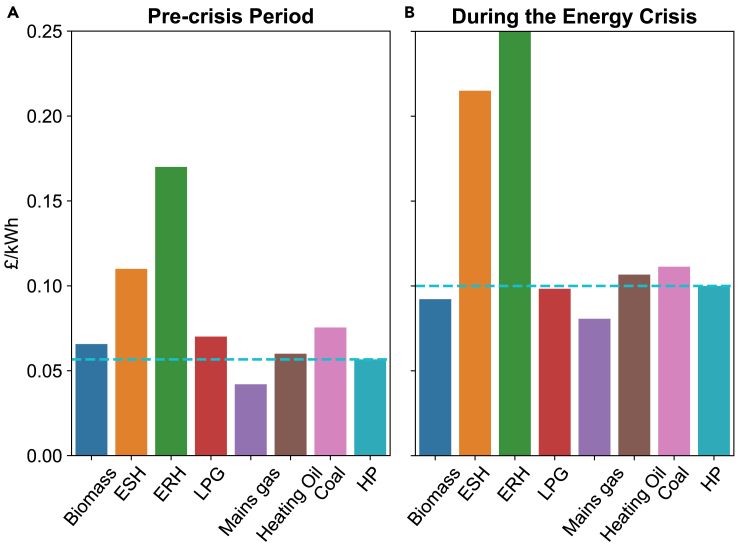
Unit heating costs of different heating options (A) Unit costs before the energy crisis. (B) Unit costs during the energy crisis. Data for electricity, gas mains, heating oil, and coal prices are obtained from BEIS.^31^^,^^32^ The prices of LPG and biofuels are based on Nottenergy.^33^ The detailed derivation of the unit heating cost is given in Section Fuel Price and Costs.

Figure 2 compares the cost per unit of heating energy of HPs with other heating options during the pre-crisis and crisis periods. The boiler efficiencies are set to those of new boilers for a like-for-like comparison, as detailed in Section Fuel Price and Costs. As illustrated, HPs have a lower running cost than all of the off-gas heating during the pre-crisis period, particularly compared to ERH and ESH. During the energy crisis, HP becomes less competitive than ‘Biomass’ and ‘LPG,’ but the HP running cost is still lower than ‘ESH,’ ‘ERH,’ ‘Heating Oil,’ and ‘Coal,’ which account for around 90% off-gas heating in England and Scotland, calculated from census data.^29^^,^^30^

#### Upfront cost

The low running cost of HPs makes it possible to see a reduced fuel poverty rate if we replace off-gas heating with HPs. However, HPs also have a much higher upfront cost than other heating options. For a typical semi-detached UK household, the upfront cost for an air-source HP without the available subsidy is £8,350, which covers the cost of the HP unit, installation, a hot water cylinder, and the necessary installation of low-temperature emitters.^15^ For HPs, there is also an option to replace the entire heating distribution system rather than just upgrading the emitter, which may lead to £6,000 more cost suggested by.^34^ The complete replacement can reduce the time to heat up the rooms.^34^ However, this is not considered a fundamental heating need and is thus not included in our fuel poverty evaluation. In comparison, the upfront cost for a combination gas boiler, including the boiler and installation, is only around £1,570-£2,250.^15^^,^^34^^,^^35^ An ESH comes with an upfront cost of £4,130, while an ERH costs around £2,500, covering the heater, installation, and hot water cylinder.^15^ A combination oil boiler has an upfront cost ranging from £1,791 to £3,560, which includes the cost of the boiler and installation.^34^^,^^35^ All of these heating methods have lower upfront costs than HPs.

**Figure 3:**
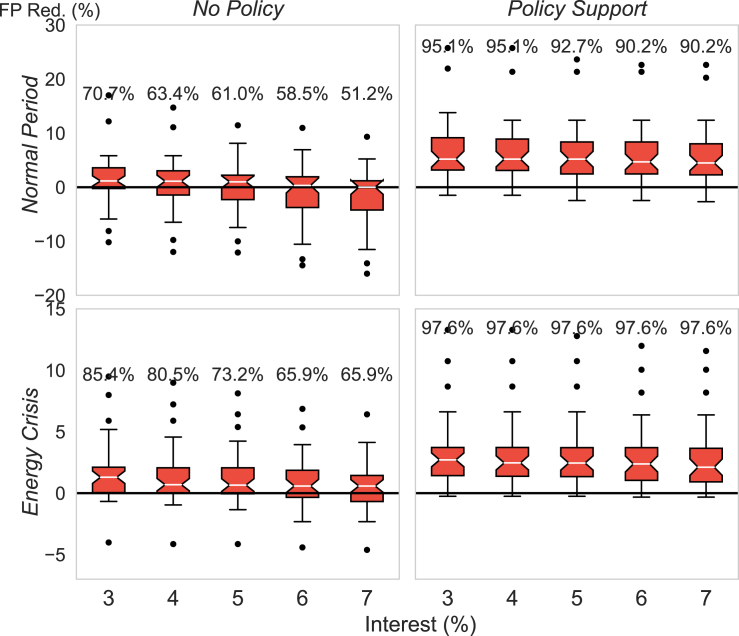
Proportion of out-of-fuel-poverty households considering the HP upfront costs Each sample (41 in total) summarized in the boxes represents the proportion of households lifted out of fuel poverty (the FP Red.) through HP replacing off-gas heating options for that region (41 England and Scotland regions in total). The first-year payment to the 20-year amortization for HP upfront cost with varying interest rates is added to the household annual fuel cost. ‘Policy Support’ means that England’s £5,000 grant is deducted from the HP upfront cost for the second column. The numbers on top of the boxes indicate the proportion of regions seeing positive fuel poverty reductions, i.e., the proportion of samples above the black horizontal lines.

The high upfront costs of HPs can dissuade residents and landlords from adopting HPs despite their low running costs. Therefore, considering the upfront cost is crucial when analyzing the economic benefits of HPs. The life expectancy of HP ranges from 15 to 25 years, according to.^36^^,^^37^ Here, we pick 20 years as an intermediate value. Amortization can be configured to distribute the cost across HP life. Note that we use the annual payment toward amortization for its better intuitiveness; however, this is equivalent to the levelized energy cost method, as explained in Section Amortization and Levelized Cost. Figure 3 displays the boxplots for the England and Scotland regions, with each sample (41 in total) summarized in the boxes representing the proportion of households lifted out of fuel poverty (the FP Red.) through HP replacing off-gas heating for that region. The numbers on top of each box represent the proportion of regions that see a positive reduction in the fuel poverty rate. The first-year payment for the 20-year amortization, considering varying interest rates for HP upfront costs, is added to the household’s annual fuel cost. We observe that even under a low interest rate of 3%, there are still 30% of regions with increased fuel poverty rates during the pre-crisis period (first row). More regions experience reduced fuel poverty rates during the energy crisis (second row) because the reduction in running costs by HPs, under higher energy prices during the crisis period, becomes more significant than their upfront costs. However, under a normal 20-year secured homeowner loan interest rate by a UK loan company (around 7%),^38^ there are still 34.1% regions with increased fuel poverty rates.

Currently the England and Scotland governments have policy support for HP uptake. In England (also Wales), the Boiler Upgrade Scheme rewards up to £5,000 (increases to £7,500 from October 2023^39^) for each household installing an air-source HP. In Scotland, the grant can cover 75% of the costs up to a maximum of £7,500, with the remainder taken as an optional interest-free loan.^40^ The second column in Figure 3 considers the England government’s £5,000 support to the HP upfront cost. A significant uplift in fuel poverty reduction rate is observed, with at least 90.2% regions seeing reduced fuel poverty rates during the pre-crisis period and 97.6% during the energy crisis, for all interest rates. Also, without policy support (the left column), there are outliers below the boxes in the plot, representing regions with high increases in the fuel poverty rate after HP replacement. With policy support, the outliers below the boxes are eliminated. These results highlight the necessity of government support in HP uptake.

In the later analysis of the paper, we will analyze the benefits of HPs replacing all the off-gas heating under the existing £5,000 England government support and assume that the household amortizes the remaining HP upfront cost for 20 years. The interest rate is set to 7%, which is close to the value provided by a UK loan company.^38^

#### Geographical illustration

**Figure 4:**
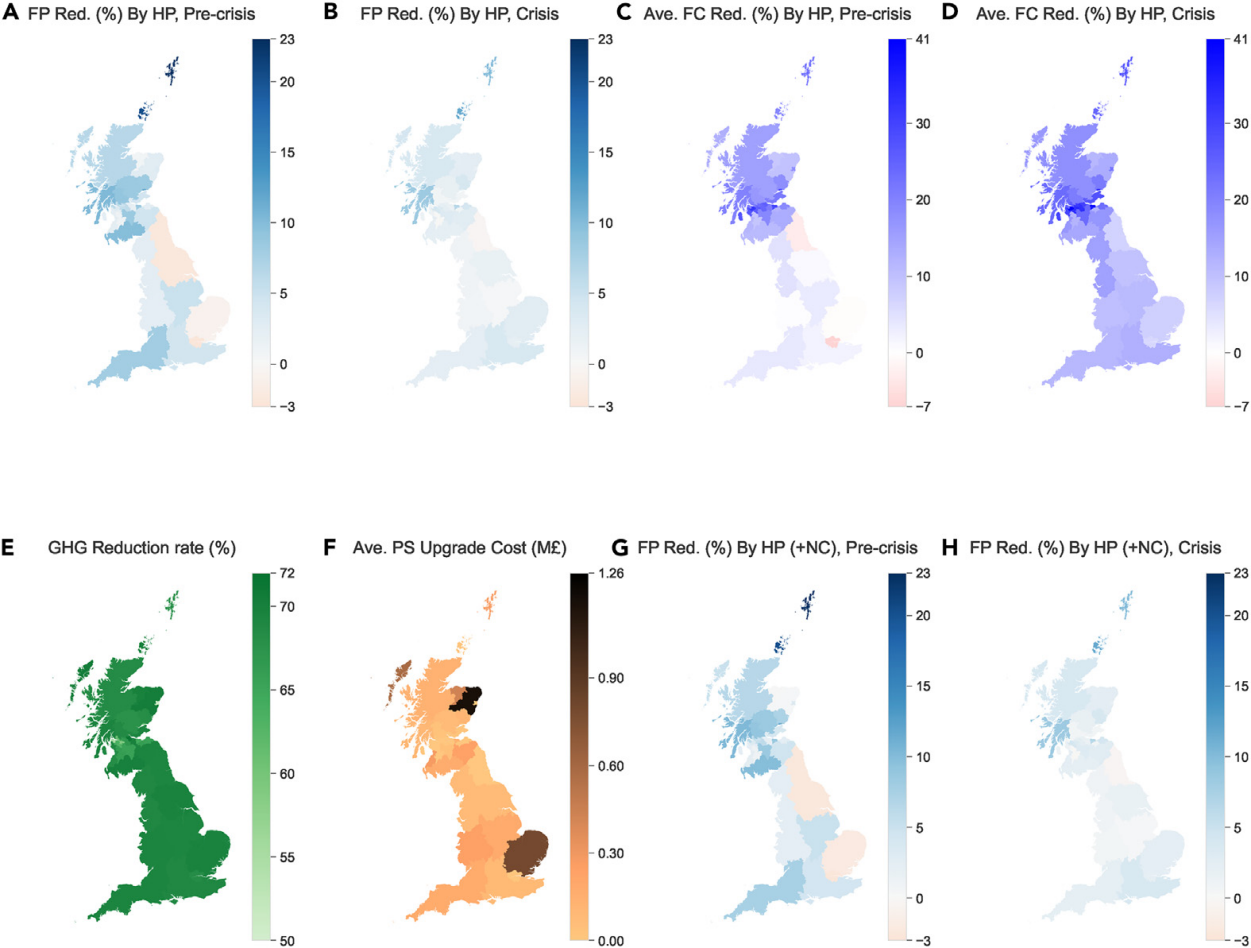
Regional fuel poverty reduction, fuel cost reduction, GHG reduction, and network upgrade costs when HPs replace off-gas heating (A) Estimated proportion of households out of fuel poverty during the pre-crisis period. (B) Estimated proportion of households out of fuel poverty during the crisis. (C) Estimated average off-gas household fuel cost reduction (FC Red) during the pre-crisis period. (D) Estimated average off-gas household fuel cost reduction (FC Red) during the crisis. (E) GHG reduction rates by region. (F) Average distribution network upgrade costs by region. (G) Estimated proportion of households out of fuel poverty considering the increased energy bills due to the network upgrade costs (highlighted as +NC) based on fuel prices pre-crisis. (H) Estimated proportion of households out of fuel poverty considering the increased energy bills due to the network upgrade costs (highlighted as +NC) based on fuel prices during the energy crisis.

We first assess the impact of the HP rollout on fuel poverty rates during the pre-crisis period. Figure 4A illustrates the results at a regional level when replacing off-gas heating with HPs. The reduction rate can be higher than 20% for specific regions.

We also estimated the fuel poverty reduction amid the energy crisis after the HP roll-out. Table 1 and Figure 4B show that the fuel poverty reduction brought by HPs becomes less significant during the crisis period. The reduction rate is only at most 11% for specific regions. We attribute the reduced effect of HPs during the crisis to fuel poverty being defined as spending above a threshold, so when a customer spends far above the threshold, even substantial reductions in their energy bills may still leave them in fuel poverty. To confirm this, we plot the average percentage reduction in off-gas household energy costs after HP replacing off-gas heating in Figures 4C and 4D. Compared to the pre-crisis period, the fuel cost reduction is even higher in the crisis period. An obvious lift is observed for the England regions, because the HP running cost reduction under a high-energy price period becomes more dominant than the amortized payment toward its upfront costs. This also means that the HP rollout indeed has a greater impact in terms of bill reduction for customers in fuel poverty, even though the impact on the fuel poverty rate is less.

#### Greenhouse gas emission reduction

Another key co-benefit of HP replacement is reduced GHG emissions in the off-gas domestic heating sector due to the lower energy consumption. We evaluated the proportion of GHG emissions that could be reduced by replacing off-gas heating with HPs. Under the GHG emission factors in the UK, 2020,^41^ GHG emissions are approximately 12.44 MT CO2e in the off-gas domestic heating sector for England and Scotland in total. When off-gas heating is replaced with HPs, GHG emissions drop to 3.86 MT CO2e, a 69% reduction.

Figure 4E provides a region-level plot of GHG emission reductions, revealing that some regions could experience a reduction above 70%. Furthermore, the majority of regions (92.7%) are expected to see GHG emission reductions of over 60%. One should note that we only consider the GHG emissions in the off-gas domestic heating sector, but the on-gas heating sector (i.e., using the natural gas network) still generates emissions. Full decarbonization of heating will require a large-scale transition, such as replacing natural gas heating with HPs or green hydrogen. Finally, although the carbon benefits of HPs have been well understood by existing literature, our analysis goes further by quantifying the extent of regional emission reduction achievable when replacing off-gas heating with HPs within the context of the current regional off-gas heating landscape and the GHG emission factors in the UK. Furthermore, by consolidating the environmental benefits and our previously demonstrated fuel poverty benefits, we can offer more comprehensive guidance to policymakers and other stakeholders who are evaluating the promotion of HPs.

### Network upgrade cost

The widespread adoption of electric HPs necessitates a reliable electricity distribution system. If the electricity demand becomes too high, the distribution system may require upgrades, which would eventually result in higher customer bills or government spending. The case is particularly severe for regions with weak local distribution infrastructure and high proportions of off-gas homes when replacing all the off-gas heating with HPs. Only 1% households used HPs as the main heating method in the winter of 2022^24^ and we hereby assume a zero HP adoption rate in the current off-gas heaters. In this paper, to estimate the whole distribution network costs triggered by the HP rollout, we start from the primary substations (PSs) since PSs are at the lowest voltage level with publicly available data for most UK DNOs. We then scale the upgrade costs of PSs to the whole distribution network based on the network cost breakdown submitted by each DNO to RIIO-ED2,^42^ assuming a stable ratio between the primary upgrade costs and the total distribution network costs. The cost breakdown by each DNO is showcased in Figure 7 and is described in Method for scaling up network upgrade cost in detail. The whole network upgrade cost estimation process is given in Figure 8 and is detailed in Method for estimating network upgrade costs.

We make a conservative estimate where all households are under the heating pattern of the Standard Assessment Procedure (SAP)^13^: they heat their home 9 h on working days and 16 h on weekends. This leads to a higher HP demand peak compared to households heating their home for a longer time with the same annual energy expenditure. More detail on the network upgrade cost estimation process is given in Section Method for estimating network upgrade costs. It is expected that 457 out of 3,891 PSs in England and Scotland will require upgrades to support the additional HP demand. The total estimated cost of these upgrades after scaling up to the whole distribution network cost is £715.4 M. For a more detailed breakdown of regional costs, Figure 4F shows the average upgrade cost (the total cost divided by the number of PSs in each region) for all regions of England and Scotland. Aberdeenshire in the top right of the map has the highest average network upgrade cost due to its relatively high proportion of off-gas homes (41.2%). East of England in the bottom right part is the second highest, although its proportion of off-gas homes in Figure 1C is relatively low (17.8%). This could be attributed to the low existing demand headroom in their distribution network, so even a small HP rollout can trigger a high cost. In particular, the two archipelagoes in the north (the Orkney and Shetland islands) with almost 100% off-gas homes still see relatively low upgrade costs. This counterintuitive result comes from the fact that more than half of households have used electric heating. Replacement of these electric heaters with HPs can be less burdensome on the network due to the high HP efficiency. The high network capacity-to-population density ratio is the other reason.

The network upgrade costs will finally be repaid by customers through their increased energy bills,^42^ which in turn affects the fuel poverty results. To consider this impact, we allocate the regional network upgrade costs to households over a 45-year period, matching the equipment lifespan,^43^ and ensuring that the net present values of additional energy bills over this period equal the total upgrade cost (see Section Customers’ repayment toward the network cost for details). We illustrate the updated fuel poverty reduction results in Figures 4G and 4H. As we can see, the HP rollout still brings substantial fuel poverty reductions to most regions after considering the additional repayments toward the network upgrades. Comparing Figures 4A and 4G, we observe reduced fuel poverty benefits over the pre-crisis period in areas with the highest network costs, specifically Aberdeenshire (reducing from 2.75% to 0.33%) and the East of England (reducing from −0.84% to −2.01%), where the latter result suggests an increase in households experiencing fuel poverty after the replacement of off-gas heaters by HPs when network costs are considered. During the crisis period (comparing 4B and 4H), there is a minimal difference, as additional energy costs due to network upgrades become relatively insignificant given the already high energy bills during the crisis. However, the reduced benefits in the pre-crisis period underscore the non-trivial impact of network upgrade costs on the long-term benefits of HPs for specific regions.

The next price control period RIIO-ED2 (from 2023 to 2028) also estimated the total distribution network upgrade cost to be £3.2 B to accommodate electric vehicles, HPs, and local low-carbon generations.^42^ This is higher than our estimated £715.4 M because (1) additional HP demand in the on-gas properties is not modeled here considering the lower economic competitiveness of HP running costs compared to mains gas, (2) we replace all the existing electric heaters in the off-gas sector with HPs, reducing the electric demand due to HP’s high efficiency, and (3) we are separating the impact of HPs given the existing distribution network headroom, separating it from network upgrades due to parallel developments like the adoption of electric vehicles. This separation aligns with our specific focus on assessing the benefits and costs associated with HPs. By keeping these factors separate, we can also attribute the additional energy costs and consequent effects on fuel poverty directly to HPs, without mixing in other contributing elements. In reality, the total network upgrades will also be triggered by other system changes, such as electric vehicle adoption and local renewable generation, which will overlap with the upgrade costs we have estimated specifically due to HPs replacing off-gas heating.

### Cost-benefit analysis

#### Maximizing benefits while minimizing costs

**Figure 5:**
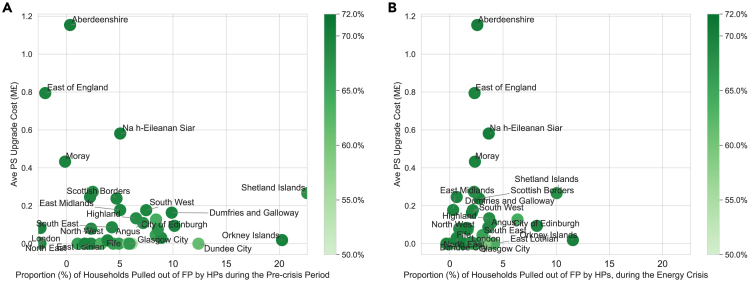
Fuel poverty (FP) reduction and GHG emission reduction versus network upgrade costs (A) The plot during the pre-crisis period. (B) The plot during the energy crisis. The colors of each plot represent the GHG emission reduction rate.

Figure 5A depicts the potential pre-crisis benefits of HP deployment, alongside the associated network upgrade costs. The fuel poverty benefits have considered the repayments toward the network upgrade costs. The plot indicates that regions closer to the right-bottom corner and marked by darker colors experience the highest benefits and lowest costs associated with HP deployment. Figure 5A reveals that most regions have relatively low network upgrade costs. In particular, certain regions such as Shetland and the Orkney Islands exhibit low costs and relatively high benefits, making them good candidates for government-led HP pilot projects and deployment initiatives. In contrast, few regions, such as Aberdeenshire and East of England, see fewer benefits and high associated costs. These results can help policymakers design and prioritize regional HP support mechanisms and deployments.

Figure 5B displays the benefits during the energy crisis period. The majority of regions see a shift to the left side due to the high energy expenditure during the crisis. However, their relative positions, such as the most ‘prior’ (Shetland and the Orkney Islands) and less ‘prior’ (Aberdeenshire and East of England), persist overall. In summary, a cost-benefit analysis can inform the government’s decision-making process regarding HP deployment, ensuring efficient and effective resource allocation to accomplish both environmental and economic objectives.

#### Multidimension correlation analysis

**Figure 6:**
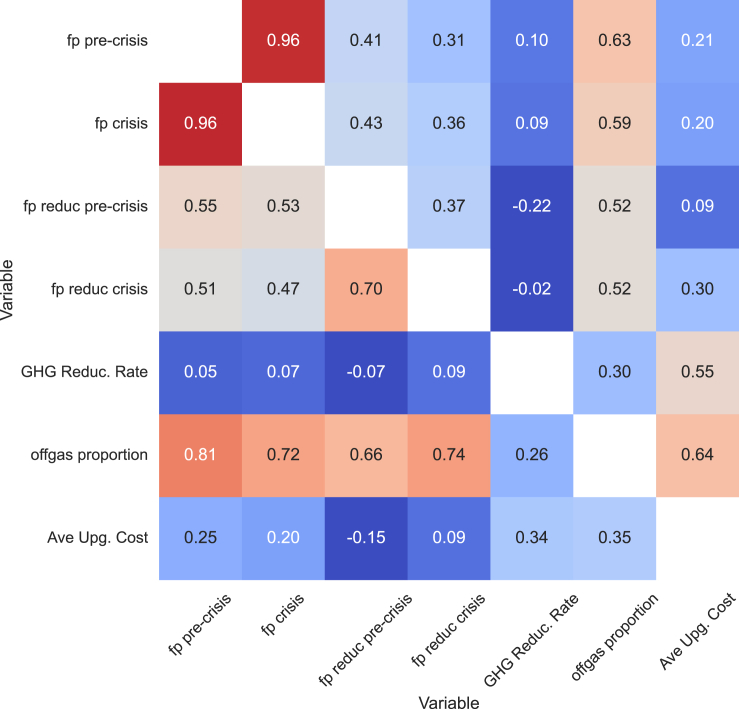
Correlation plot for the central dimensions of our study These dimensions include the estimated fuel poverty (fp) and reduction rates before and during the energy crisis periods, GHG emission reduction rates, distribution network upgrade costs, and off-gas proportion. The lower triangle plot is based on Pearson correlation for a linear relationship while the upper triangle is based on Spearman’s coefficient that captures a nonlinear relationship.

Figure 6 presents an extensive correlation graph, examining the relationships among the key dimensions of our study, namely the fuel poverty estimates and the reduction rates before and during the energy crisis periods considering the repayments toward the network upgrade costs triggered by HPs, GHG emission reduction rates, distribution network upgrade costs, and the proportion of off-gas households. The lower triangle plot is based on the Pearson correlation for linear relationships, while the upper triangle is based on Spearman’s coefficient, capturing nonlinear relationships. The plot indicates that regions with high fuel poverty rates are highly likely to experience even higher rates during the energy crisis, motivating policy interventions in these vulnerable areas. The off-gas proportion exhibits a strong positive correlation with both the fuel poverty rate and the fuel poverty reduction by HPs during both periods. This result highlights the vulnerability of highly off-gas regions but also a stronger positive effect by HP roll-out, which may be worth a prioritized deployment of more affordable and sustainable heating alternatives, such as HPs. The high linear correlation (0.7) between fuel poverty reduction by HPs in pre-crisis and crisis periods also suggests that HPs could be a robust solution for mitigating fuel poverty.

We do not observe a linear correlation (close to 0) between network upgrade costs and fuel poverty reduction in both periods. Spearman’s correlation results (0.30) suggest a positive but weak monotonic relationship in the crisis period. This result indicates that higher economic benefits may not necessarily come with higher network upgrade costs. Indeed, regions such as Shetland and Orkney Islands enjoy a high reduction in fuel poverty and low network upgrade costs, as discussed in Sections Maximizing benefits while minimizing costs and Network upgrade cost. Although there is a positive Spearman correlation (0.55) between the network upgrade cost and GHG reduction rate, the variation in GHG reduction across regions, as depicted in Figure 4E, is not substantial. These findings highlight that the higher cost is not necessarily attached to the higher benefits of HP replacement at the regional level.

It is essential to acknowledge the distinction between correlation and causation. Our analysis primarily identifies the correlation among different dimensions but does not establish causality. More specific analysis and experiments are needed to interpret the causal relationship between various factors.^44^

## Discussion

HP has a low running cost but a high upfront cost. Taking the upfront cost into consideration leads to even higher fuel poverty rates for many regions when we replace off-gas heating with HPs. Government support is necessary, and we show that the economic benefits of HP replacement can be restored with the England government grant. Meanwhile, with the massive production of HPs in the future, HP may enjoy a lower upfront cost, making it more economically viable. Another approach to increase the economic benefits of HPs can be the integration of local renewable energy sources (RES), such as local solar or wind generation. An online survey carried out in the UK^45^ shows that only 33.2% households would not consider installing solar panels at their home, demonstrating the feasibility of local RES deployment. Private-owned generation may alleviate the electricity cost and therefore the cost of HP heating. Local RES can also be installed for an entire community or shared among households to reduce the upfront cost, which can be incentivized by an appropriate local energy market (LEM) design that fairly distributes cost and profits.^46^^,^^47^

A recent study^28^ concluded a negative impact on fuel costs when replacing natural gas heating with HPs, even without considering the upfront cost of HPs. Our Figure 2 has a similar observation that HPs have a higher running cost than natural gas. Our case studies illustrate that the economic benefits of HPs hold for off-gas households.

In addition to the £5,000 (increases to £7,500 from October 2023^39^) government subsidy for HP installations, the UK has introduced several policies to address fuel poverty, such as the Energy Bills Support Scheme, Energy Price Guarantee, and subsidies for energy efficiency measures. A valuable avenue for future research is to determine which of these policies has the most substantial impact on reducing fuel poverty. Addressing this question can guide the development of more effective policies for alleviating fuel poverty using public funds. A related approach was demonstrated in a study by ref.^48^, where the fuel poverty metric was used to plan targeted energy interventions.

HPs can be flexible in terms of the ability to be turned on/off for a short period while maintaining residents’ comfort. This flexibility makes HP capable of engaging in the local flexibility market (LFM). LFM is an emerging technology that UK DNOs are adopting,^49^ where energy flexibility can be traded to provide services to the power system and bring profits to end-users.^50^ The previously mentioned LEM and LFM here with different time horizons can also be stacked for HP engagement. The Local Energy Oxfordshire (LEO) project is considering and evaluating the market value stack in the Oxfordshire area of the UK.^51^ LEO is also testing the flexibility provision of smartened HPs in Oxfordshire residents.^52^ However, it is also worth noting that HPs may have smaller flexibility than other heating options: the high upfront cost of HPs makes the installed thermal power usually smaller than other heating options,^34^ leading to a slower indoor temperature adjustment.

In addition to economic barriers, air source HP installation can also cause significant disruption due to the need to install a bulky fan unit,^53^ which may be less acceptable to some residents. Finally, for large-scale HP roll-out, there could be insufficient HP production or installers. If these supply chain issues persist, the fuel poverty reduction brought about by HPs during the energy crisis period may not appear as evident, as the natural gas price is projected in^54^ to return to normal by 2030. Nevertheless, geopolitical tensions and uncertainties indicate that gas prices will continue to be volatile, arguably more so than the increasingly renewable-intensive electricity.^55^ A range of policies is also now in place to address these issues and accelerate HP deployment. These policies include the Boiler Upgrade Scheme to attract public investment in HPs, the “Heat Pump Investment Accelerator Competition” to support component manufacturing infrastructure across the UK, and the “Heat Training Grant” to increase the number of qualified HP installers, etc.^56^^,^^57^ We expect that these policy measures can, to some extent, expedite the HP rollout process. Furthermore, while the benefits are contingent on supply chain and installation processes, the initial step is to acknowledge the potential benefits that HPs can offer to address the fuel poverty issue, which is the primary focus of our study. Lastly, our findings demonstrate that HP benefits endure in both normal and crisis periods. This means that even if the crisis ends before HP adoption reaches a substantial level of impact, the adoption of HPs can still yield long-term benefits, particularly considering the possibility of future energy crises.

Building efficiency is also important for HP deployment. Although building fabric is less likely to affect the HP efficiency,^58^ poor insulation will require larger-size HPs, resulting in higher upfront costs. Rural regions are likely to have this building efficiency issue, hindering the financial benefits of HPs. However, similar to the capital costs of solar and wind energy which have fallen 40% and 80% respectively during the past 10 years,^28^ we anticipate a lower upfront cost of HPs with the development of HP markets, thereby alleviating the additional HP upfront cost in less-efficient buildings.

The wide-range HP roll-out can threaten the electric distribution network as analyzed in Section Network Upgrade Cost. As an alternative option for heating electrification, ESH has the advantage of being able to shift demand to off-peak periods, thus alleviating the burden on the power grid. HPs can also be equipped with additional thermal storage, alleviating the burden on the power grid like ESH and also saving energy bills by charging on a low-tariff period.^53^ Storage can also offer HPs with higher demand flexibility, which facilitates more stable power system operation and brings more economic benefits to HP owners by participating in demand response and various LEM and LFM.^12^^,^^59^ The integrated HP and storage system is recognized as a key to consumer demand satisfaction and a stable electricity grid by the International Energy Agency.^60^

Note that HP is not always a burden on the power grid. For regions where electric resistive heating is being massively adopted, replacing the resistive heating with HPs can even lead to lower electric demand because of the high efficiency of HPs. Our analysis in Section Network Upgrade Cost has already illustrated this, where the two off-gas-grid archipelagoes (the Orkney and Shetland Islands) with high electric resistive heating adoption enjoy a low upgrade cost after HP replacement.

Our estimated distribution network upgrade costs are scaled from the PS level due to the limited data availability. The more precise analysis would require detailed network information that is protected by the corresponding DNOs.

The rebound effect is an extensively discussed fact that could lead to actual energy saving lower than expected.^61^ One example is that households may consume more energy than before after the energy efficiency is improved. This effect may lead to energy consumption reduction and GHG emission reduction by HPs less than simulated. The current fuel poverty definition in the UK seems to avoid this issue as the fuel poverty indices are calculated based on the modeled energy consumption, rather than those actually consumed. However, the peak electricity demand and thus the network upgrade cost could be higher, which may still lead to higher modeled fuel costs through the increased electricity price to repay DNOs and thus lower fuel poverty benefits.

The UK has significant socio-economic inequalities, especially between the north and south.^28^ For example, it is shown that productivity, measured as income divided by working hours, is on average lower in the northern regions than southern regions. The pandemic has exacerbated this issue. Our results have illustrated that the benefits and costs brought by HPs are not evenly distributed among the Scotland and England regions. Consequently, it is crucial to exercise prudence and prioritize equitable outcomes during the HP deployment process, aligning with the core objectives of the UK Leveling Up program,^62^ which seeks to address existing inequalities.

### Limitations of the study

We acknowledge the following limitations of our work. First, this study applied the 10% definition of fuel poverty. Scotland’s metric additionally considered the remaining income after deducting the fuel costs, and England’s definition includes the “low fuel poverty energy efficiency rating”. Some energy bill discounts, such as the Warm Home Discount used in the official fuel poverty estimations, were not considered in this study. Therefore, our estimated fuel poverty results could deviate from the actual official fuel poverty estimations. Justifications for the 10% definition and a detailed comparison with the current official metrics in the UK are presented in Method for Fuel Poverty Estimation. Future work can adjust the metrics and make new definition through the process in Method for Fuel Poverty Estimation with necessary data. Note that the economic benefits of HPs for off-gas homes should persist as the energy cost reduction is independent on the fuel poverty definition.

Second, our estimated distribution network costs serve as an indicator of the potential network upgrades and could also deviate from the actual network upgrade costs. Accurately estimating network upgrade costs is complicated and requires more comprehensive information regarding the distribution network.

## STAR★Methods

### Key resources table

**Table:** 

REAGENT or RESOURCE	SOURCE	IDENTIFIER
**Deposited data**		

Scottish House Condition Survey	UK Data Service^63^	UK Data Service: https://doi.org/10.5255/UKDA-SN-8974-1
English Housing Survey	UK Data Service^64^	UK Data Service: https://doi.org/10.5255/UKDA-SN-8891-1
Prices of electricity and gas	Department for Business, Energy and Industrial Strategy, Quarterly energy prices^31^	https://www.gov.uk/government/statistical-data-sets/annual-domestic-energy-price-statistics
Prices of heating oil and coal	Department for Business, Energy and Industrial Strategy, Consumer prices index^32^	https://www.gov.uk/government/statistical-data-sets/monthly-domestic-energy-price-stastics
Prices of LPG and biofuels	Nottingham Energy Partnership^33^	https://nottenergy.com/resources/energy-cost-comparison/
Per-kWh GHG Emissions by Fuels	Department for Energy Security and Net Zero, Green-house gas reporting: conversion factors 2020^41^	https://www.gov.uk/government/publications/greenhouse-gas-reporting-conversion-factors-2020
Scotland census data	National Records of Scotland^29^	https://www.scotlandscensus.gov.uk/search-the-census#/
England census data	Office for National Statistics^30^	https://www.ons.gov.uk/census
Primary substation information in SP Energy Networks	SP Energy Networks^65^	https://www.spenergynetworks.co.uk/pages/long_term_development_statement.aspx
Primary substation information in Scottish & South Electricity Networks	Scottish & South Electricity Networks^66^	https://network-maps.ssen.co.uk/
Primary Substation information in Western Power Distribution	National Grid^67^	https://www.nationalgrid.co.uk/our-network/network-capacity-map-application
Primary substation information in UK Power Networks	UK Power Network^68^	https://ukpowernetworks.opendatasoft.com/explore/dataset/ukpn_primary_postcode_area/information/
Primary substation information in Electricity North West	Electricity North West^69^	https://www.enwl.co.uk/get-connected/network-information/long-term-development-statement/
Primary substation information in Northern Power Grid	Northern Power Grid^70^	https://www.northernpowergrid.com/demand-availability-map
Normalised heater demand profile	A. Canet, M. Qadrdan, N. Jenkins, J. Wu (2022)^71^	https://data.ukedc.rl.ac.uk/browse/edc/efficiency/residential/Buildings/heat_demand_by_local_area/
Case study outputs	This paper	https://github.com/EsaLaboratory/Datasets--Social-Benefits-and-Network-Costs-of-Heat-Pumps

**Software and algorithms**		

Software	Python	https://www.python.org/
Codes for reproducing the analysis in this paper	GitHub	https://github.com/EsaLaboratory/Codes--Social-Benefits-and-Network-Costs-of-Heat-Pumps

### Resource availability

#### Lead contact

Further information and questions regarding this manuscript should be directed to and will be fulfilled by the lead contact, Yihong Zhou (yihong.zhou@ed.ac.uk).

#### Materials availability

This study did not generate new unique materials.

#### Data and code availability

• The source data is available as listed in the key resources table. More specifically, we used the Scottish House Condition Survey (SHCS) and English Housing Survey (EHS) datasets to derive the household information as described in Household Dataset. Fuel prices are also necessary for estimating the fuel cost (see Fuel Price and Costs). The price data used in this paper includes 1) prices of electricity and gas, 2) prices of heating oil and coal, and 3) prices of LPG and biofuels, coming from three different sources in the UK. Data for per-kWh GHG emissions by fuels were also used in this study to estimate the regional GHG emission, with the estimation method detailed in Section Method for Estimating Greenhouse Gas Emissions. Scotland census and England census provide the household proportion by each heating fuel type at a regional level, which facilitates the calculation of the number of heaters for each primary substation as described in upgrade cost estimation process. The primary substation information in each UK DNO is necessary to estimate the network upgrade cost as detailed in Method for Estimating Network Upgrade Costs. These pieces of information are obtained from each of the corresponding DNOs respectively. The normalised heater demand profile was also used to calculate the peak network demand as described in upgrade cost estimation process.

Finally, to facilitate subsequent research and provide a better reference, we stored our case study outputs in a CSV file and placed it on GitHub. The publicly accessible link is listed in the key resources table. This file has 41 rows, corresponding to the 32 Scotland LAs and 9 England government regions studied here. The stored case study output file includes our fuel poverty estimation before and after HPs replacing off-gas heating, GHG emission reduction, and network upgrade costs. A detailed description for each row and column is associated with the CSV file and can be accessed through the link mentioned above.

• Codes for reproducing the analysis in this paper are publicly placed on GitHub and the link is listed in the key resources table.
• Any additional information required to reanalyze the data reported in this work paper is available from the lead contact upon request.

### Method details

#### Household dataset

Two national surveys, the English Housing Survey (EHS) and the Scottish House Condition Survey (SHCS), provide processed household interview data in.^64^^,^^63^ The SHCS dataset^63^ spans from 2012 to 2019 (inclusive) and includes approximately 3,000 samples per year. Each sample represents an anonymous household and contains information on:

• Household adjusted net income: the income of all adults in the household after deduction of the income tax payable on that income, the national insurance contributions, and housing costs.^3^
• Modelled energy consumption: the required energy in kWh for the household for one year, including energy for heating, cooking, and lighting.
• Primary heating fuel type: the primary heating fuel for this household. Examples include mains gas, electricity, heating oil, LPG, etc.
• Local Authority (LA): the LA where this anonymous household locates.
• Sampling weights: the weight is applied to each household interview sample, so the weighted statistics of the whole dataset can be representative of the whole nation.

This information is sufficient to calculate the 10% fuel poverty rate (described in Section Fuel Poverty Definition) for each region. We use data from 2017 to 2019 to obtain a more accurate estimation for each LA, as recommended in their methodology report; otherwise, the lowest number of data samples for a single LA would be only 54.

The English dataset follows a similar format, and we use the most recent data from 2019, which contains approximately 12,000 samples. The minimum number of samples per region is 729, so there is no need to include multiple years as in the case of Scotland. Unlike the Scottish data, which links each sample to a specific LA, each sample in the English dataset only indicates the corresponding government region, out of nine total. This classification (which is less granular than LAs) is the lowest geographical level for our England fuel poverty analysis results. The regions, formerly known as the government office regions, are the highest tier of sub-national division in England, which includes South East, London, North West, East of England, West Midlands, South West, Yorkshire and the Humber, East Midlands, and North East.

#### Method for fuel poverty estimation

This section first introduces the fuel poverty definition used in this study and then showcases the methodology for estimating fuel cost, a key component in fuel poverty calculation.

##### Fuel poverty definition

This study uses the “10% definition” of fuel poverty: A household is considered to be in fuel poverty if its necessary fuel cost is more than 10% of the household adjusted net income.

Official definitions of fuel poverty vary in the UK. Wales applies the 10% definition like us and considers a household in fuel poverty if its necessary fuel cost is more than 10% of the household’s total income.^72^ In Scotland, the household’s income is differently defined as the household adjusted net income and there is one more condition for a household considered fuel-poor: the household’s remaining adjusted net income is insufficient to maintain an acceptable standard of living.^3^ The fuel poverty definition in England was also the 10% definition before 2012, and it changed after that to include the “low fuel poverty energy efficiency rating” as a condition to consider fuel-poor.^14^

Compared to the Scottish definition, the “10% definition” of fuel poverty used in this study may consider rich households spending a lot of energy as fuel-poor, but from another perspective, the results based on this can also help rich households save their energy bills, meaning that the 10% definition here can be indicative for all levels of income compared to the Scottish definition that pays more attention to low-income households. The consideration of the household efficiency rating as in the current English definition is less relevant to our study, as our focus is mainly on the benefits of the impact of high-efficiency heating technology rather than the housing stock on fuel poverty.

##### Fuel price and costs

Electricity and mains gas prices are sourced from BEIS quarterly energy prices,^31^ while heating oil and coal prices are obtained from the BEIS Consumer Price Index.^32^ Both datasets represent national statistics. LPG and biofuel prices are derived from Nottenergy,^33^ which collects price information from suppliers within the East Midlands region of England. England’s fuel poverty data specifies three categories of heating fuels: 1) mains gas, 2) electricity, and 3) other. As the fuel poverty dataset does not specify whether the electric heating is ERH or ESH, we assume the price for ‘electricity’ option as the weighted average price of ESH (5/11) and ERH (6/11), based on their share in UK household electric heating surveyed by BEIS.^11^ ESH benefits from the UK Economic-7 tariff, and we follow^15^ and assume that 90% of the daily consumed energy is charged over 0:00 and 7:00 am, the low-price period in Economic-7. We define the fuel price for “other" as a weighted average of all non-gas fuels (excluding electricity), with weights determined by the household heating proportions in England based on 2021 England census data.^30^ Note that one unit of heating energy may require more than one unit of fuel due to the heater’s efficiency. We set the heater efficiency at 90% for gas, LPG, and oil boilers, 75% for coal boilers, 100% for electric heating, and 300% for HP, as suggested in.^15^^,^^33^ The unit heating cost $c_{u}$ in Figure 2 is finally calculated as the unit fuel price divided by the heating efficiency. Additionally, fuel costs in fuel poverty calculations include cooking, lighting, and other costs of energy usage, all of which are assumed to have standard electricity tariffs $c_{e}$ in this study. Given the heating cost setting, we calculate the fuel cost for each household as

(Equation 1) $$\text{Fuel}\;\text{cost}\;£=c_{u}\times E_{\text{sw}}+c_{e}\times E_{\text{other}}$$

where $E_{\text{sw}}$ refers to the energy consumption in kWh for space and water heating and $E_{\text{other}}$ refers to other energy consumption including cooking and lighting. The unit heating cost $c_{u}$ is determined by the primary heating type of the household.

We do not consider fixed costs, such as standing charges for metered fuels or maintenance and delivery costs for non-metered fuels, as our primary focus is on energy usage costs. Moreover, our study centres on HP replacements for off-gas heating, which means that considering fixed costs would essentially make HP a more economical option, as off-gas non-electric heating fixed costs would be reduced (almost all properties use electricity, so they already need to pay the electricity standing charge). The estimated annual maintenance cost is £90-£125 for gas boilers, £100-£180 for oil boilers, and £80-£125 for LPG boilers.^73^ The maintenance cost for HP can be around £175, which is slightly more expensive, but the gap can be wiped out by not paying the fixed cost for off-gas non-electric after HP replacement.^15^ Government benefits like the Warm Home Discount (WHD) are also not included for the same reason.

The EHS dataset only provides the modelled energy cost (£) rather than energy consumption (kWh). We estimate the corresponding energy consumption for heating each household in kWh using the heater efficiency and the fuel price information in the same year of the England dataset (2019) as below:

(Equation 2) $$\text{Energy}\;\text{consumption}\;\text{kWh}=\frac{C_{\text{sw}}}{\text{Unit}\;\text{heating}\;\text{cost}\;2019}$$

where $C_{\text{sw}}$ is the modelled cost for space heating and water heating in the dataset, and the unit heating cost is determined by the main heating option of the household.

Finally, when evaluating the fuel poverty status of a household using an HP, we will add the first-year payment towards the amortised HP upfront cost to their annual fuel cost. We can obtain the first-year payment under varying interest rates from an online calculator^74^ or the formula in.^75^

##### Amortisation and levelised cost

Fuel poverty by definition is determined by the annual fuel cost. Therefore, we use the first-year payment towards the amortisation as this is intuitive and directly reflects the additional payment for each year in Section Upfront Cost. However, this is essentially equivalent to the calculation by the levelised lifetime cost method, which is widely used in measuring the per-unit energy cost considering capital investments.^76^ We illustrate the equivalence below:

Denote $C^{u}$ the upfront cost of an HP, $E^{y}$ the annual energy consumption (kWh), *P* the unit fuel cost (£/kWh), *N* the expected life-span, *r* the annual interest/discount rate. The levelised lifetime cost LC (£/kWh) can be calculated as:^76^

(Equation 3) $$LC=\frac{C^{u}+\sum_{n=1}^{N}\frac{E^{y}\times P}{{\left(1+r\right)}^{n}}}{\sum_{n=1}^{N}\frac{E^{y}}{{\left(1+r\right)}^{n}}}$$

where the maintenance cost is not considered as discussed in our Section Fuel Price and Costs. The end-of-life cost is not considered as well for simplicity. Note that LC is usually calculated on an annual basis. Here *n* starts from 1 to account for the cash flow at the end of each year.

The annual energy bill $C^{yu}$ considering the upfront cost can be derived by scaling up the levelised cost LC, i.e.,

(Equation 4) $$\begin{array}{cc} C^{yu} & =E^{y}\times LC\\ & =E^{y}\times \frac{C^{u}+\sum_{n=1}^{N}\frac{E^{y}\times P}{{\left(1+r\right)}^{n}}}{\sum_{n=1}^{N}\frac{E^{y}}{{\left(1+r\right)}^{n}}}\\ & =E^{y}\times \frac{C^{u}}{\sum_{n=1}^{N}\frac{E^{y}}{{\left(1+r\right)}^{n}}}+E^{y}\times P\\ & =\frac{C^{u}}{\sum_{n=1}^{N}\frac{1}{{\left(1+r\right)}^{n}}}+E^{y}\times P\\ & =\frac{C^{u}}{\left(1-{\left(\frac{1}{1+r}\right)}^{N}\right)/r}+E^{y}\times P\\ & =\frac{C^{u}r{\left(1+r\right)}^{N}}{{\left(1+r\right)}^{N}-1}+E^{y}\times P \end{array}$$

where the second term $E^{y}\times P$ is the annual running cost, while the first term $\frac{C^{u}r{\left(1+r\right)}^{N}}{{\left(1+r\right)}^{N}-1}$ is exactly the amount of amortisation paid under an annual cash flow.^75^ In many real-world cases the amortisation cash flow is monthly which is used in our calculation, while the levelised cost is usually calculated yearly.^76^ However, they are essentially equivalent.

#### Method for estimating greenhouse gas emissions

The domestic heating of a household generates an amount of GHG emissions that can be calculated by:

(Equation 5) Emissions=Fuelconsumptionforheat×GHGfactor

where the fuel consumption for heat is calculated as heating energy divided by the heater efficiency introduced in Section Fuel Price and Costs. The per-kWh GHG emissions by combustion comes from the Department for Energy Security and Net Zero, Greenhouse gas reporting.^41^ Note that we focus on the GHG emissions caused by domestic heating only, excluding other household activities that generate GHG emissions.

#### Method for estimating network upgrade costs

The widespread adoption of electric HPs could trigger upgrades for distribution network infrastructures, which can be a financial burden for DNOs and eventually result in higher customer bills. We start with Primary Substations (PS), which is a crucial part of the UK distribution network and is also at the lowest voltage level with publicly available data for most UK DNOs. We then scale the upgrade costs of PSs to the whole distribution network as detailed in section method for scaling up network upgrade cost.

##### Substation dataset

DNOs in the UK publish PS information on their corresponding websites^65^^,^^66^^,^^67^^,^^68^^,^^69^^,^^70^ listed in the key resources table. Our required information includes 1) existing peak demand (net of local generation contribution), 2) firm capacity in MVA, 3) power factor (PF) that, informally speaking, measures how much electrical power can be actually consumed by end-users, 4) longitude and latitude which are used to infer their corresponding geographical regions. The information may be contained in single or multiple files for a DNO. We extract and merge all the information and get our processed PS dataset. Entries with missing information except for PF are dropped, resulting in 4210 out of 4347 PSs, an approximately 3.15% reduction. Where PF is missing, it is set to 0.9855, the average of the substations with PF. As we specifically focus on England and Scotland, we extract the PSs in these two countries, and there are 3,891 PSs in total.

##### Upgrade cost estimation process

The flowchart for estimating the network upgrade cost triggered by HPs replacing off-gas domestic heating is given in Figure 8. For the $i^{\text{th}}$ PS, its upgrade cost $C^{\text{PS},i}$ to accommodate the new demand of HPs is calculated as:

(Equation 6) $$C^{\text{PS},i}=Cap^{\text{ex},i}\times C^{u}$$

where $Cap^{\text{ex},i}$ is the exceeding capacity in MVA (the amount of electrical power that a PS cannot supply). $C^{u}$ is the unit cost of each MVA upgrade of a PS derived in (vii). The exceeding capacity $Cap^{\text{ex},i}$ of a PS can be further calculated as:

(Equation 7) $$Cap^{\text{ex},i}={\bar{D}}^{\text{new},i}-Cap^{\text{PS},i}$$

where ${\bar{D}}^{\text{new},i}$ is the new peak demand after the HP rollout (section new PS peak demand) for this PS, and $Cap^{\text{PS},i}$ is the firm capacity of the PS described in section firm capacity.

In the following, we describe how the new PS peak demand is derived based on the bottom-up order, as shown in the flowchart. The description focuses on the $i^{\text{th}}$ PS of a region (the minimum region unit in our fuel poverty estimation), and we assume this region has a total $N^{\text{PS}}$ PSs. One can easily repeat the calculation for each PS and make a summation to get the regional PS upgrade cost.

###### Number of connected households

The first step is to calculate the number of connected households $N^{h,i}$ for the $i^{\text{th}}$ PS we are evaluating. The exact number of $N^{h,i}$ is not provided for most of the UK DNOs, and here $N^{h,i}$ is estimated by dividing the total number of households $N^{h}$ in this region by the PSs’ existing peak demand ${\bar{D}}^{i}$ from the PS dataset, which implicitly neglects the effect of other demand than the domestic sector, e.g., industrial sector. In other words,

(Equation 8) $$N^{h,i}=N^{h}\times \frac{{\bar{D}}^{i}}{\sum_{i}^{N^{\text{PS}}}{\bar{D}}^{i}}$$

As a toy example, suppose region A with 10,000 households has two PSs: PSa and PSb. The peak demand in PSa is 4 MW, while that in PSb is 6 MW. Then based on our method, there will be 4,000 households connected to PSa and 6,000 to PSb.

###### Number of HPs and existing electric heaters

This step calculates the number of required HPs to replace all the off-gas heaters $N^{\text{HP},i}$, i.e., the number of off-gas heaters, and the number of existing electric heaters $N^{\text{EH},i}$ connected to the $i^{\text{th}}$ PS.

An ideal approach would be to first identify the supply area of each PS and then combine this information with census data on household heating proportions. However, most DNOs do not disclose the exact supply regions in a processable format. Based on the estimation in section number of connected households, the mean and median number of connected households for PSs in England are 7445 and 6320 respectively. For Scotland PSs, the mean and median are 3185 and 2864 respectively. In census data, the geo-levels that are closest to the number of PS-level households are Middle Layer Super Output Area (MSOA) for England and Intermediate Zone (IZ) for Scotland. The mean and median number of households for an MSOA are 3418 and 3308. The mean and median household numbers of an IZ are 1855 and 1802. Therefore, a PS can roughly support two MSOAs or two IZs in England or Scotland.

This observation suggests that the household heating component for PS *i* can be derived by combining the household heating component data for the two nearest MSOAs in England and the two nearest IZs in Scotland (available in Census^29^^,^^30^). For $N^{\text{HP},i}$, by knowing the number of off-gas heaters in the two nearest MSOAs/IZs denoted as $N^{\text{HP},i,1}$ and $N^{\text{HP},i,2}$, we have:

(Equation 9) $$N^{\text{HP},i}=N^{h,i}\times \frac{N^{\text{HP},i,1}+N^{\text{HP},i,2}}{N^{h,i,1}+N^{h,i,2}}$$

where $N^{h,i}$ stands for the total number of connected households of the $i^{\text{th}}$ PS estimated in section number of connected households. $N^{h,i,1}$ and $N^{h,i,2}$ are the total number of households in the two nearest MSOAs/IZs from the census dataset. The number of existing electric heaters $N^{\text{EH},i}$ can be calculated in the same manner.

For example, consider a PS estimated to supply 10,000 households. The two closest IZs are IZ1 and IZ2. IZ1 has a total of 5,000 households, with 1,000 using off-gas heating and 300 using electric heating. IZ2 also has 5,000 households, 2,000 of which use off-gas heating and 700 using electric heating. In this case, the number of HPs for PS is calculated as 10,000 × (1,000 + 2,000) / (5,000 + 5,000) = 3,000, and the number of existing electric heaters is 10,000 × (300 + 700) / (5,000 + 5,000) = 1,000.

Note that the fuel poverty datasets also have information on the number of households in each heating option, but for the region (LA for Scotland and Government Regions for England) in total. For better consistency, we scale the estimated number of various heaters for each PS so that the summed number of heaters of all the PSs for this region matches the fuel poverty dataset.

###### New HPs and existing electric heating demand profiles

An open dataset in^71^ provides a typical normalised HP electricity demand profile, with a one-year duration and half-hour resolution. This demand profile is denoted as a vector $D^{\text{HP},u}=\left[D_{1}^{\text{HP},u},\cdots ,D_{t}^{\text{HP},u},\cdots ,D_{17520}^{\text{HP},u}\right]$. Here “normalised” means the one-year demand profile sum up to 1 kWh of electricity usage, i.e., $\sum_{t}D_{t}^{\text{HP},u}=1$. This demand profile incorporates the After Diversity Maximum Demand (ADMD), allowing for a more accurate peak demand estimate when multiple HPs are connected to a PS.

For the $i^{\text{th}}$ PS, we calculate the total new HP heating profile $D^{\text{HP},i}$ by

(Equation 10) $$D^{\text{HP},i}=N^{\text{HP},i}\cdot \frac{E^{\text{heat}}}{\mu^{\text{HP}}}\cdot D^{\text{HP},u}$$

where $N^{\text{HP},i}$ is the number of required HPs to replace all the off-gas heaters in section number of HPs and existing electric heaters. $E^{\text{heat}}$ is the mean household heating energy for the region, as can be determined from the fuel poverty dataset (Section Household Dataset). $\mu^{\text{HP}}$ is the efficiency, which is the Seasonal Performance Factor (SPF) for HPs here. In this study, we assume a PF of 1 for HPs as suggested by,^77^ which means that HPs do not contribute reactive power.

The dataset also provides a normalised ERH demand profile. As in our analysis of fuel poverty in Section Fuel Price and Costs, the existing electric heating demand is calculated as 6/11× ERH demand +5/11× ESH demand. The ESH demand is derived by reassigning the ERH demand so that the energy consumed between 0:00 and 7:00 am accounts for 90% of the daily consumed energy. We calculate the total demand profile $D^{\text{EH},i}$ contributed by existing electric heating in the same way as $D^{\text{HP},i}$.

###### PS existing demand profile

The PS dataset in Section Substation Dataset contains PS peak active power demand ${\bar{D}}^{P,i}$, which is then used to scale the normalised UK total electricity demand data published and processed in.^78^ The demand profile for the year 2019 is selected because this is the latest year with full-year data. We get the one-year existing electric active power demand profile $D^{P,i}$ for the PS after scaling. The reactive power demand profile $D^{Q,i}$ can be calculated as

(Equation 11) $$D^{Q,i}=D^{P,i}\cdot \sqrt{\frac{1-\alpha^{2}}{\alpha^{2}}}$$

where α is the PF recorded in the substation datasets.

###### New PS peak demand

Given 1) the new HP demand profiles $D^{\text{HP},i}$ due to replacing off-gas heaters in section new HPs and existing electric heating demand profiles, 2) the existing electric heating demand profile $D^{\text{EH},i}$ in section new HPs and existing electric heating demand profiles, and 3) existing active and reactive demand profiles $D^{P,i}$ and $D^{Q,i}$ in section PS existing demand profile, the new yearly demand profile $D^{\text{new}}$ (apparent power in MVA) of the $i^{\text{th}}$ PS can be calculated as:

(Equation 12) $$D^{\text{new}}=\sqrt{{\left(D^{Q,i}\right)}^{2}+CLIP{\left(D^{P,i}+D^{\text{HP},i}-D^{\text{EH},i}\right)}^{2}}$$

where $D^{P,i}+D^{\text{HP},i}-D^{\text{EH},i}$ represents the new active power demand profile, and the negative sign in $-D^{\text{EH},i}$ is because the existing electric heating (ESH and ERH) is also replaced with HPs. The CLIP function is to set negative elements to zero. In our case, the new active power demand profiles can contain negative values for 0.155% of time-steps for all PS in total throughout the year. This could be due to the inaccuracy of the estimated number of existing electric heaters for each PS.

The new peak demand ${\bar{D}}^{\text{new},i}$ after the HP rollout is calculated as:

(Equation 13) $${\bar{D}}^{\text{new},i}=\max_{D_{t}^{\text{new}}\in D^{\text{new}}}D_{t}^{\text{new}}$$

###### Firm capacity

Based on a UK DNO Northern Powergrid,^70^ firm capacity $Cap^{\text{PS},i}$ refers to the capacity of a substation that is immediately available following the occurrence of a first circuit outage. Power system operation has a high requirement on reliability and resilience, so firm capacity can well measure if a PS can supply electricity over peak time under extreme conditions. This information has been included in our processed PS dataset as described in Section Substation Dataset.

###### Unit upgrade cost

We obtained several PS upgrade proposals from a UK DNO, which provided the installed new capacity and the associated cost. By averaging these, we determined the upgrade cost per exceeding MVA $C^{u}$ to be £236,900/MVA. It is important to note that this is only a high-level estimation which aims to provide insights and indications rather than a precise figure which would depend on local network details. A detailed analysis of PS upgrade cost is complex and requires a granular evaluation by the corresponding DNO.

Finally, based on new peak demand ${\bar{D}}^{\text{new},i}$ after the HP rollout in section new PS peak demand, the firm capcity $Cap^{\text{PS},i}$ in section firm capacity, and the unit upgrade cost $C^{u}$ in section unit upgrade cost, we can calculate the exceeding capacity $Cap^{\text{ex}}$ through Equation 7 and then the upgrade cost $C^{\text{PS},i}$ of the $i^{\text{th}}$ PS through Equation 6.

##### Heating patterns

The heating boilers may be turned off when residents are away from their homes. The two fuel poverty datasets used in this study take this behaviour into account when modelling households’ required heating energy, resulting in two categories of heating patterns:

• 9 hours on working days (6-8:00 am and 17-24:00) and 16 hours on weekends (8-24:00), which is the heating pattern in SAP.^13^
• 16 hours (8-24:00) every day, considering that a household member stays at home daily to care for children or has vulnerable members.

The heating season is assumed to span from October to May. These heating pattern assumptions are used to derive our heating demand profiles for consistency. Note that, as the exact household composition connected to each PS is unavailable, we assume that all households follow the first heating regime, i.e., 9 hours on weekdays and 16 hours on weekends. Given the same amount of heating energy, a reduction in the heating period will lead to an increase in heating demand, leading to a conservative estimate of network upgrade costs.

##### Method for scaling up network upgrade cost

**Figure 7:**
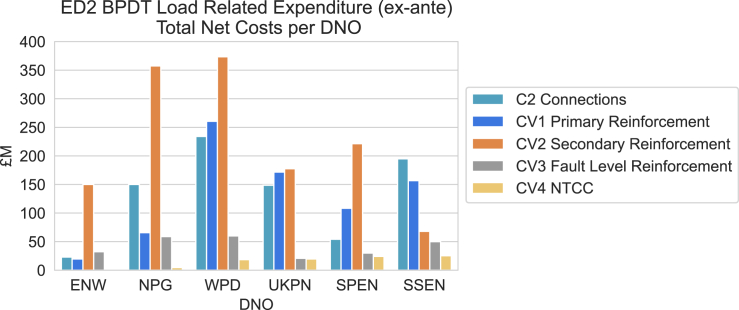
Breakdown of load related expenditure by distribution network operators (DNOs) extracted from RIIO-ED2 Draft Determinations 2022

To estimate the distribution network upgrade costs triggered by HPs replacing off-gas heating, we start from PSs since they are at the lowest voltage level with publicly available data for most UK DNOs. Based on Figure 7 from the core methodology document of RIIO-ED2 Draft Determinations,^42^ the primary reinforcement only accounts for a relatively small proportion of the total Load Related Expenditure (LRE) for the distribution grids. Assuming the ratio between the primary network costs and the total network costs obtained from Figure 7 are stable, we can scale our estimated primary network upgrade costs to the total distribution network costs triggered by HPs replacing the off-gas heating for each DNO.

**Figure 8:**
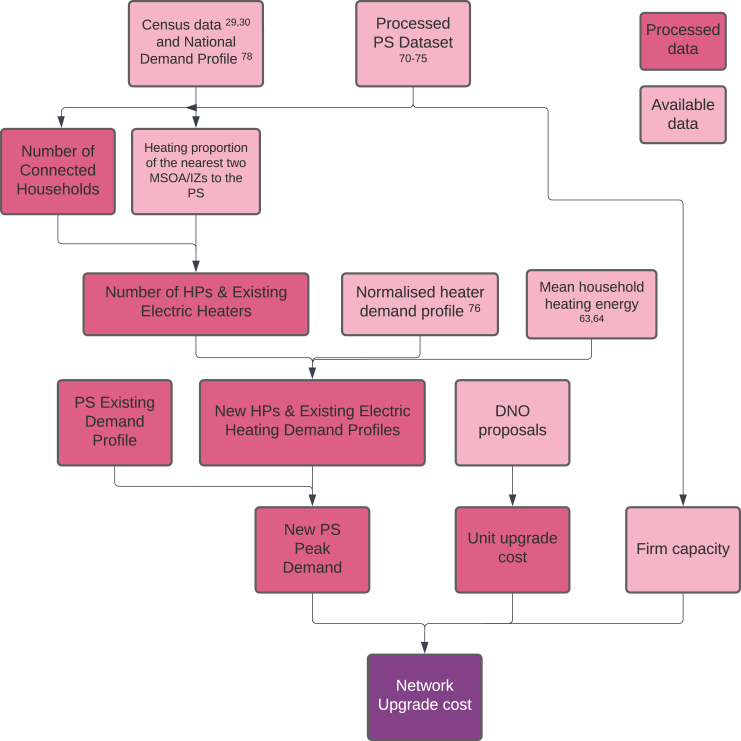
Flowchart for estimating the distribution network upgrade costs

##### Customers’ repayment toward the network cost

Customers will finally repay the network upgrade costs through their increased energy bills,^42^ which in turn affects the fuel poverty results. To consider this impact, we follow the same setting in^43^ to spread the network costs over the 45-year lifespan of upgraded network equipment. In addition, because the future money is worth less, the Net Present Value from the additional customers’ bills should equal the total cost of the network upgrade $c^{net}$ for that region:

(Equation 14) $$c^{net}=N\sum_{i=0}^{45}\frac{c^{a}}{{\left(1+r\right)}^{i}}$$

where $c^{a}$ is the additional annual energy bill for one household, *N* is the total number of households in that region, and *r* is the discount rate. We set it to 7%, the interest rate under a normal 20-year secured homeowner loan interest rate by a UK loan company,^38^ which is also used to spread out the HP upfront cost over its life span.

The additional energy bill $c^{a}$ per household per year can be therefore calculated as follows:

(Equation 15) $$c^{a}=\frac{c^{net}}{N\sum_{i=0}^{45}\frac{1}{{\left(1+r\right)}^{i}}}$$
